# Resveratrol: Potential Application in Sepsis

**DOI:** 10.3389/fphar.2022.821358

**Published:** 2022-02-09

**Authors:** Jiajia Li, Xiaoting Zeng, Fuxun Yang, Lan Wang, Xiaoxiu Luo, Rongan Liu, Fan Zeng, Sen Lu, Xiaobo Huang, Yu Lei, Yunping Lan

**Affiliations:** Department of ICU, Sichuan Provincial People’s Hospital, University of Electronic Science and Technology of China, Chengdu, China

**Keywords:** sepsis, resveratrol, organ protection, antioxidation, SIRT-1, animal model

## Abstract

Sepsis is a life-threatening organ dysfunction syndrome caused by host response disorders due to infection or infectious factors and is a common complication of patients with clinical trauma, burns, and infection. Resveratrol is a natural polyphenol compound that is a SIRT-1 activator with anti-inflammatory, antiviral, antibacterial, antifungal inhibitory abilities as well as cardiovascular and anti-tumor protective effects. In recent years, some scholars have applied resveratrol in animal models of sepsis and found that it has an organ protective effect and can improve the survival time and reduce the mortality of animals with sepsis. In this study, Medline (Pubmed), embase, and other databases were searched to retrieve literature published in 2021 using the keywords “resveratrol” and “sepsis,” and then the potential of resveratrol for the treatment of sepsis was reviewed and prospected to provide some basis for future clinical research.

## Introduction

Sepsis develops following a dysregulated host immune response in patients with clinical trauma, burns, and infection leading to organ dysfunction. Progression to septic shock leads to multiple organ dysfunction syndromes, which is the primary cause of death in critically ill clinical patients, with a mortality rate reaching up to 30%–70% (Singer et al., 2016). The pathogenic factors and mechanisms contributing to sepsis are multifactorial and complex, including epigenetic and transcriptional regulation disorders, neuroendocrine-immune network disorders, coagulation abnormalities, tissue and organ damage, as well as inflammatory metabolic damage and microbial toxin effects (Singer et al., 2016; van der Poll et al., 2017; Cecconi et al., 2018). Therefore, it has been challenging to identify an effective agent for the prevention and treatment of sepsis that can address all of these factors while having a good safety profile.

Resveratrol (C_14_H_12_O_3_, molecular weight 228.25) is a polyphenol antitoxin produced by plants in response to exogenous stimuli, such as ultraviolet light, mechanical damage, or fungal infection (Sanders et al., 2000). Resveratrol is widely distributed in plants’ roots, stems, leaves, and fruits and is well known for its potent antioxidant activity. Its name is derived from the roots of white hellebore, where it was first identified in 1940 (Shakibaei et al., 2009) and has since been widely identified in grapes, knotweed, peanuts, mulberries, blueberries, spruce, and other plant roots, leaves, and fruits (García-Pérez et al., 2012). Polyphenols inhibit NF-κB activation and downregulate nitric oxide synthase, adhesion molecules, and tumor necrosis factor-α. The expression of polyphenols and the enhancement of endogenous antioxidant capacity may also contribute to the effectiveness of polyphenols, which can effectively improve sepsis-related organ damage (Shapiro et al., 2009). Resveratrol is an effective Sirtuin-1 (SIRT-1) (Arunachalam et al., 2010) activator with anti-inflammatory (Zimmermann-Franco et al., 2018), antiviral (Campagna and Rivas 2010), antibacterial, and antifungal inhibitory properties (Lu et al., 2008). It also possesses cardiovascular and anti-tumor protective effects (Shang et al., 2019) (Ashrafizadeh et al., 2021).

In recent years, some scholars have applied resveratrol in animal models of sepsis. The application of resveratrol in endotoxemia rats can reduce the occurrence of oxidative damage by inhibiting erythrocyte lipid peroxidation and catalase (CAT) activity, reducing nitric oxide (NO) release, downregulating malondialdehyde (MDA) levels, and maintaining iron homeostasis (Sebai et al., 2009). Resveratrol can induce increased activation of AMPK in macrophages stimulated by lipopolysaccharide (LPS) via the Ca^2 +^/CaMKKβ pathway. This leads to protection against bacterial infections by increasing phagocytosis, regulating inflammatory status, and inhibiting the development of endotoxin tolerance by inhibiting the expression of IRAK-M and SHIP-1 induced by LPS (Quan et al., 2021). Recent studies have found that resveratrol also protects organ function during sepsis in various ways. This paper summarizes its organ protective effects and application potential in sepsis and offers direction for future research based on the results of various studies (Table 1).

**TABLE 1:** Application of resveratrol in different organs of sepsis models.

Organ or system	Dosage	Pathway or mechanism	References NO.
Lung	40 mg/kg	↑VEGF-B; ↓ TNF-α、IL-6 、 IL-1β	Yang et al. (2018)
MH-S cell	10 μM	↑VEGF-B siRNA; ↓ NF-κB phosphorylation、ERK1/2 and p38; ↓ Bax ↑ Bcl-2; ↓ LC3-II/Iratio; ↓ C5aR; ↑C5L2	Yang et al. (2018)
Lung	0.3 mg/kg	↓MDA and H2O2; ↑GSH/GSSG ratio、T-AOC、CAT and SOD activity; ↓iNOS and NO; ↓Peroxynitrite	Zhang et al. (2014)
Lung	30 mg/kg	↓PI3K/Nrf2/HO-1 pathway	Wang et al. (2018b)
Lung	60 mg/kg	↓JAK2/STAT3 pathway	Ji et al. (2016)
Lung/Kidney	30 mg/kg	↓MDA, ↑GSH; ↓Collagen content; ↓TNF-α and LDH activity	Kolgazi et al. (2006)
Heart	30 mg/kg	↑PI3K/AKT/mTOR pathway	Shang et al. (2019)
Heart	10 ul/g	↓TNF-α 、IL-1β 、MIP-1α 、MCP; ↑Nrf2, ↑ gene HO-1 and GCLM expression	Hao et al. (2013)
Myocardial Cells	3 µM	↓capase 3 activation; ↓ROS; ↑ Nrf2 Activation	Hao et al. (2013)
Heart	20 mg/kg	↓MDA; ↑SOD and POD; ↓CAT; ↓NO	Sebai et al. (2011)
Heart	20 mg/kg	↑SERCA2a activation	Bai et al. (2016)
Heart	30 or 60 mg/kg	↑PGC-1 mRNA、Protein expression and transcriptional activity	Smeding et al. (2012)
Heart	60 mg/kg	↓TNF-α and MPO; ↑ Sirt1 and Bcl-2 expression; ↓ Ac-FoxO1 and Bax expression	An et al. (2016)
Heart	60 mg/kg	Activation of Sirt1 signal, ↓ neutrophil aggregation, TNF-α expression and myocardial cell apoptosis	An et al. (2016)
Kidney	10 mg/kg	↓NO; Improve microcirculation	Holthoff et al. (2012)
Kidney	15 mg/kg	↓ TLR4-NF-κB pathway	Chen et al. (2015)
Kidney	15 ml/kg	↓NF-κB Activation; ↓Er stress	Luo et al. (2020)
Kidney	30 mg/kg	↓IRE1-NF-κB pathway	Nian Wang et al. (2017)
Kidney	50 mg/kg	Recovery of SIRT1/3 activity, ↓ acetylation of SOD2, ↑GSH, GSH/GSSG ratio and CAT activity; ↑ATP content, ↓ mPTP opening	Xu et al. (2016)
Kidney	3, 10, 30, 100 mg/kg	↑renal capillary perfusion, RBC velocity, and blood flow	Holthoff, et al. (2011)
Kidney	100 mg/kg	↓TNFα, IL-1β, IL-6 and McP-1; ↓Renal vascular permeability; ↑IL10; ↓Bcl-2 and Bcl-XL	Chen, et al. (2015)
BMDM	10, 20, 50, 100 µmol	↓TLR4-NF-κB pathway	Chen, et al. (2015)
BV2	15 or 30 µM	↓NLRP3, caspase - 1 and IL - 1β	Sui et al. (2016)
Liver	60 mg/kg/d	↓HMGB1 Cytoplasmic translocation	Xu et al. (2014)
Brain	10 and 30 mg/kg	↓apoptosis; ↓ IBA-1; ↓NLRP3 and IL-1 β	Sui et al. (2016)
Brain	30 μM	↓ NLRP3 and IL-1 β cracking	Sui et al. (2016)
Brain	8 mg/kg/d	↓MMP-9protein, ↓Occludin and Claudin-5 degradation	Liu et al. (2020)
Circulation	30 mg/kg	↓Total blood viscosity and local blood flow	Wang et al. (2018a)
Circulation	30 mg/kg	↑ RhoA-ROCK-MLCPpathway	Xu-Qing Wang et al. (2017)
Circulation	5 mg/kg and 10 mg/kg	↓Rac-1 and HIF-1α; ↑eNOS expression	Zhang et al. (2019)
Circulation	30 mg/kg	↓White cell/platelet adhesion,↓ E - element/ICAM 1; ↑SIRT1; ↓E-selectin/ICAM-1	Xianfeng Wang et al. (2015)
Immune	1, 5, 10, 20, or 40 µM	↓ TNF-αand IL-6, ↓MAPK and STAT)1/STAT3; ↑SOCS1; ↓ miR-155 expression	Ma et al. (2017)
Immune	50 μM	↓ TRAF6 expression and ubiquitination, ↓ TLR4-TRAF6; ↓ MAPK and Akt pathway	Jakus et al. (2013)
Immune	30, 50 μM	↓ TRIF-TBK1-RIP1 pathway	Youn et al. (2005)
Immune	10–500 lM	↓ TNF-α、IL-1β、IL-6、MCP-1、MIP1α和HMGB-1; ↓ NF - κB activation, SphK activity and ERK1/2 phosphorylation	Sebai et al. (2010)
Immune	1 mg/kg	↓ PLD, SphK1, ERK1/2 and NFκB signaling molecules Activation	Wang et al. (2020)
Immune	100 mg/kg	↓ DNA damage of lymphocytes	Aydın et al. (2013)
Immune	50 or 300 μM	↓ Ca2 +/CaMKK β pathway↓, AMPK activation↓ IRAK-M and SHIP1	Quan et al. (2021)
Gastrointestinal Tract	100 mg/kg	↓ TNFα and IL-6; ↓ Ileum smooth muscle reaction	Gacar et al. (2012)
Adrenal Gland	0.3 mg/kg	↓ iNOS, NO, peroxynitrite; Left the MDA; ↑T-AOC, CAT and SOD activities	Duan et al. (2016)

## Materials and Methods

Two authors searched the literature published in 2021 through MEDLINE (PubMed), EMBASE, and other databases. Using “resveratrol” and “sepsis” as keywords, they included the literature meeting the following criteria: patients with sepsis and animal models of sepsis were studied; studies were clinical trials or animal experiments of resveratrol intervention; and the primary endpoints were changes in organ function, circulatory status, and inflammatory response. Three authors conducted this study in three stages: analyzing the title followed by the abstract and, finally, reading the full text in detail. They were able to retrieve 77 articles from PubMed and 181 from EMBASE; 144 irrelevant articles were excluded by title, 41 were duplicate articles (114 in two databases), and four systematic reviews were excluded after reading the abstracts. One meeting abstract, three non-English studies, 17 non-sepsis studies, and 14 low-quality literature pieces were also excluded. Finally, 34 studies were included after reading the full text. These included basic studies, including animal experiments and cell experiments, on the effects of resveratrol on the lung, heart, kidney, liver, brain, adrenal gland, gastrointestinal function, and circulatory and immune systems of sepsis models.

## Effects of Resveratrol on Various Organs/Systems in Sepsis Models

### Lung

**FIGURE 1:**
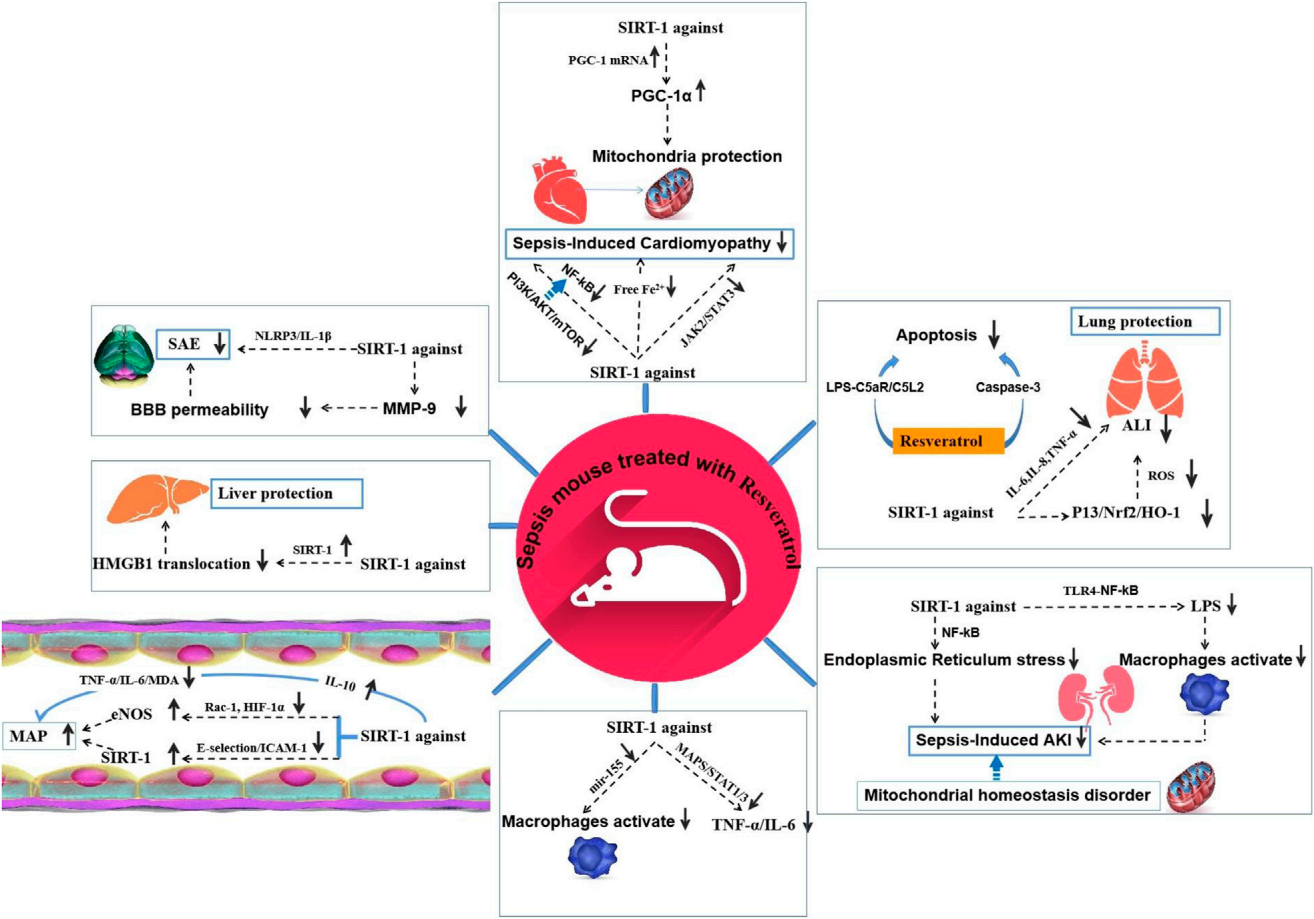
Effection of Resveratrol on different organs of sepsis models.

The lung is the most readily injured organ in sepsis, and acute lung injury is one of the first manifestations of sepsis with the highest incidence among affected organs (Rubenfeld et al., 2005; Matthay et al., 2019). Yang et al. (2018) demonstrated that resveratrol treatment significantly reduced acute lung injury induced by cecal ligation and puncture (CLP) in mice (Figure 1). This protective effect could be attributed to the ability of resveratrol to modulate the autophagy and anti-apoptotic effects of C5aR and C5L2 induced by LPS. Moreover, resveratrol can reduce the expression levels of inflammatory factors associated with the response to infection, such as tumor necrosis factor (TNF)-α, interleukin (IL)-6, and IL-1β, by inhibiting the vascular endothelial growth factor-B pathway. In addition, resveratrol pretreatment (Zhang et al., 2014) inhibited the expression of induced nitric oxide synthase (iNOS) and the production of NO caused by endotoxemia. It also significantly reduced the formation of peroxynitrite in lung tissues. These findings support the therapeutic potential of resveratrol in reducing acute lung injury caused by oxidative/nitrification processes.

Moreover, resveratrol downregulates inflammatory mediators (IL-8, RANTES, IL-1α, IL-6, TNF-α, and CXCL10) and has specific inhibitory effects on acute lung injury caused by respiratory viruses. *In vitro* studies suggest that caspase-3 levels in infected cells treated with resveratrol reduce virus-induced apoptosis (Filardo et al., 2020). Severe acute respiratory syndrome coronavirus 2 (SARS-CoV-2) infection can strongly induce the release of cytokines and chemokines resulting in cytopathy and organ dysfunction. Resveratrol inhibits excessive inflammation and oxidative responses in elderly COVID-19 patients by activating Nrf2 in aging vascular smooth muscle cells to reduce the production of mitochondrial reactive oxygen species (ROS) (Liao et al., 2021). Resveratrol may also play a positive role in sepsis-related injuries caused by viruses. In a rat model of sepsis, it was found that the use of resveratrol can reduce the levels of MDA and myeloperoxidase in lung and kidney tissues and increase the content of glutathione. This effect balances the oxidant–antioxidant state and reduces the oxidative damage of lung and kidney tissues (Kolgazi et al., 2006). At the same time, a previous study showed that timely administration of resveratrol after the occurrence of sepsis could reduce the degree of acute lung injury in septic rats by inhibiting inflammation, oxidative stress, and apoptosis via the inhibition of the PI3K/Nrf2/HO-1 signaling pathway (Wang et al., 2018b). These data further support the view that resveratrol plays an active role in the Nrf-2 signaling pathway (Farkhondeh et al., 2020).

### Heart

Sepsis-induced cardiomyopathy is a common complication of sepsis. Approximately 50% of sepsis patients will suffer from myocardial injury to varying degrees, and the mortality rate of sepsis patients with such complications is approximately 80% (Flierl et al., 2008). The pathogenesis of sepsis-induced cardiomyopathy is extremely complex and remains in an early exploration stage despite extensive efforts. Recent studies have shown that the occurrence of sepsis-induced cardiomyopathy is the result of a variety of factors including myocardial inhibition; mitochondrial dysfunction; oxidative stress; and imbalance of calcium regulation, apoptosis, and adrenoceptors (L’Heureux et al., 2020). Shang et al. used resveratrol as an intervention for septic myocarditis in a rat model, showing protection of the septic myocardium by activating the PI3K/AKT/mTOR signaling pathway and inhibiting the NF-κB signaling pathway and related inflammatory factors (Shang et al., 2019). In addition, Hao et al. (2013) revealed that resveratrol preconditioning can inhibit LPS-induced ROS production by activating the Nrf2 pathway in a rat myocardial injury model *in vivo* and in primary cultured human cardiomyocytes *in vitro*. At the same time, the use of resveratrol in the CLP sepsis rat model decreased the sepsis-induced cardiomyocyte apoptosis and reduced the inflammatory cytokine TNF-α in serum and IL-1β in myocardial tissues. It also inhibited activation of the Janus activated kinase 2 (JAK2)/signal transducer and activator of transcription 3 (STAT 3) pathway, thereby reducing myocardial damage (Ji et al., 2016).

Sebai et al. (2011) also found that resveratrol can antagonize LPS-induced lipid peroxidation, decrease the activity of superoxide dismutase (SOD), and reverse the increase in myocardial NO production induced by LPS. More importantly, resveratrol could reduce the LPS-induced decrease in myocardial free iron and reduce overall cardiotoxicity.

The systematic inflammatory cascade associated with sepsis can cause myocardial contractile dysfunction by impairing the calcium response (Shankar-Hari et al., 2016a; Shankar-Hari et al., 2016b). Resveratrol improved cardiac dysfunction in an LPS-induced endotoxemia mouse model. The mechanism for this protective effect was attributed to enhanced sarcoplasmic endoplasmic reticulum Ca^2+−^ATPase activity by promoting the oligomerization of phospholamban (Bai et al., 2016). Peroxisome proliferator-activated receptor gamma coactivator 1 (PGC-1) is a coactivator of nuclear transcription that plays a key role in regulating the activity of many types of nuclear receptors. Among them, PGC-1α, the first and most well-studied signaling pathway, is the transcriptional coactivator of mitochondrial-related genes and participates in regulating of mitochondrial biosynthesis and function. The PGC-1α signaling pathway can promote the synthesis of myocardial mitochondria, induce energy production, maintain the contractile and diastolic function of the myocardium, and enhance the ability of cardiomyocytes to resist oxidative damage (Karunakaran et al., 2015). In another study (Smeding et al., 2012), resveratrol (30 mg/kg or 60 mg/kg) was subcutaneously injected into the neck of a CLP sepsis model, which caused an increase in PGC-1 mRNA and protein expression as well as transcriptional activity and further improved the mitochondrial integrity of the heart tissue compared with that of the control group as assessed with electron microscopy.

As one of the most extensively studied pathways of the cardiovascular system, the SIRT-1 pathway is highly sensitive to the cellular redox state and resists ROS through the deacetylation of various of cells, thus protecting and maintaining the vascular function of the heart (Nadtochiy et al., 2011a; Nadtochiy et al., 2011b; Vinciguerra et al., 2012; Hwang et al., 2013). Resveratrol effectively activates the SIRT-1 pathway as well. SIRT-1 activation improves mitochondrial function, increases ATP production in cells, and improves cellular metabolism (Price et al., 2012). Through the SIRT-1 pathway, resveratrol can exert a wide range of anti-inflammatory effects leading to beneficially therapeutic outcomes in inflammatory diseases (Dai et al., 2018). SIRT-1 can also control endothelial homeostasis and vascular function by regulating the expression of endothelial nitric oxide synthase (eNOS) activity, p53, and angiotensin II (Ang II) type 1 receptor (AT1R) (Kitada et al., 2016).

In the rat model of CLP-induced myocardial injury, intraperitoneal injection of resveratrol (An et al., 2016) was able to reduce myocardial injury during sepsis by decreasing neutrophil accumulation, producing the myocardial cytokine TNF-α, activating the SIRT-l pathway, reducing the production of myeloperoxidase, and suppressing cardiomyocyte apoptosis.

### Kidney

Overall, 36% of critically ill patients in the intensive care unit suffer from acute kidney injury (AKI) during hospitalization (Bagshaw et al., 2009). Among AKI patients without severe underlying diseases, the mortality rate can reach up to 10%–20% (Hoste et al., 2018). It has been reported that 40%–70% of AKI cases in the United States are caused by sepsis (Fani et al., 2018), and the mortality rate of septic AKI (SAKI) is significantly higher than that of AKI or sepsis alone (Bagshaw et al., 2009). The pathogenesis of SAKI has not yet been fully elucidated, although inflammation, oxidative stress, microvascular endothelial dysfunction, and renal tubular epithelial cell injury have all been proposed to play a role.

When LPSs enter the body, the LP-binding protein binds to CD14 and becomes an endotoxin monomer, which is transferred to the copolymer formed by TLR4 and MD2 adaptor protein, activating the TLR4 receptor to, in turn, activate the NF-κB pathway, ultimately increasing the production of pro-inflammatory factors, such as IL-6, and aggravating the inflammatory response of the kidney tissue. In the early stage of sepsis, renal microvascular disorders are related to the production of active nitrogen. As an effective polyphenol vasodilator, resveratrol protects renal tubular epithelial cells by improving renal microcirculation and eliminating the dual mechanism of active nitrogen species, thus reducing renal injury in sepsis (Holthoff et al., 2011). In addition, LPS-induced iron mobilization from the plasma to the kidney could be eliminated by resveratrol treatment. These results suggest that resveratrol has a strong antioxidant effect on LPS-induced nephrotoxicity, and its mode of action seems to be related to the iron shuttle protein (Holthoff et al., 2012).

Chen et al. also demonstrated that resveratrol can effectively regulate the activation of LPS-stimulated macrophages via the TLR4-NF-κB signaling pathway, thereby reducing the inflammatory response (Chen et al., 2015). In addition, Luo et al. (2020) showed that resveratrol could reduce renal injury in a septic rat model by inhibiting the activation of NF-κB and reducing endoplasmic reticulum stress. Subsequently, Nian Wang et al. (2017) found that resveratrol treatment immediately after successful establishment of the LPS-induced sepsis model could inhibit the phosphorylation of inositol demand enzyme 1 (IRE1) and NF-κB in the kidney. This conclusion was further supported with *in vitro* models suggesting that resveratrol can prevent septic AKI mainly by inhibiting renal inflammation triggered by the IRE1-NF-κB pathway. It was also found in another animal experiment that resveratrol can reduce LPS-induced cytokine production, decrease the concentrations of IL-1β, IL-6, McP-1, and TNF-α in plasma and kidney, and decrease the renal tubular vacuole changes and pathological apoptosis (Chen et al., 2015).

In recent years, the role of mitochondrial dysfunction in SAKI pathogenesis has received increasing attention. Resveratrol is a chemical SIRT-1 activator that can effectively restore SIRT-1/3 activity, reduce the level of acetylated SOD2 (ac-SOD2), improve oxidative stress and mitochondrial function of renal tubular epithelial cells, and prolong survival time. Mice with renal injury and sepsis showed decreased SIRT-1/3 activity as well as increased ac-SOD2 levels, oxidative stress, and mitochondrial damage. All of these parameters improved following resveratrol treatment. This suggests that the protective effect of resveratrol on renal function may depend on SIRT1-mediated SOD2 deacetylation to maintain mitochondrial homeostasis (Xu et al., 2016).

### Liver

Sepsis-induced liver injury is often undetected and neglected in clinical settings despite this injury substantially increasing the risk of death from sepsis. Therefore, there is an urgent need to clarify the mechanism of sepsis-induced liver injury and to identify new therapeutic targets to improve the survival outcomes of these patients. In acute liver failure, high-mobility group protein-1 (HMGB1) translocation from the nucleus to the cytoplasm increases (Zhou et al., 2011) and inhibition of HMGB1 secretion alleviate systemic inflammatory response syndrome and sepsis-induced organ damage (Wang et al., 2008). Conversely, the release of HMGB1 from hepatocytes increases the risk of liver damage during sepsis. Therefore, inhibition of the translocation and release of HMGB1 can potentially prevent liver injury during sepsis and may provide a wider treatment window. Sepsis-induced serum transaminase activity and pro-inflammatory chemokine levels were decreased by resveratrol pretreatment, which also improved the liver histological parameters associated with the upregulation of SIRT-1. Knockout of SIRT-1 *in vitro* further confirmed that resveratrol increased the inhibition of SIRT-1-mediated HMGB1 translocation (Xu et al., 2014).

### Brain

Sepsis-associated encephalopathy (SAE) is characterized by brain dysfunction associated with sepsis, and its mechanisms include the production of inflammatory cytokines, microscopic brain injury, blood-brain barrier damage, changes in brain metabolism, changes in nerve transmission, and disruption of the brain microcirculation (Gofton and Young 2012). Microglia are highly activated in SAE, depending on injury to the nerve, humoral pathway, or blood-brain barrier (Gofton and Young 2012). In LPS-exposed astrocytes, resveratrol upregulates adenosine receptor expression and exerts anti-inflammatory effects by inhibiting NFκB and p38 mitogen-activated protein kinase (p38 MAPK) through the activation of Nrf-2/HO-1, SIRT-1, and PI3K/Akt pathways (Bobermin et al., 2019). In a study done by Sui et al. (2016), SAE was induced in mice by CLP; mice treated with resveratrol at 10 and 30 mg/kg demonstrated better spatial memory during water maze training compared to those in the control group. Moreover, resveratrol effectively inhibited the increase in NLRP3 expression and IL-1β cleavage in a dose-dependent manner. Subsequent *in vitro* experiments in the BV2 microglial cell line showed that resveratrol prevented ATP-induced NLRP3 activation and IL-1β cleavage, which was reversed by treatment with the SIRT-1 inhibitor nicotinamide. These findings suggested that the protective effect of resveratrol on mice SAE is achieved by regulating the NLRP3/IL-1β pathway and that the increase in blood-brain barrier permeability during sepsis is a key link in the occurrence and development of SAE (Varatharaj and Galea 2017). Liu et al. injected 12-week-old male rats with 8 mg/kg resveratrol twice daily for 2 days, which inhibited the expression of matrix metalloproteinase-9 protein in cortical astrocytes, thus reducing the degradation of occludin and claudin-5 tight junction proteins to strengthen the blood-brain barrier, and finally reduced the degree of cognitive dysfunction caused by SAE (Liu et al., 2020).

### Circulation

Sepsis-related microcirculation failure is widely observed in clinical settings. It involves complex pathogenesis including the uncontrolled release of inflammatory mediators that contribute to sepsis itself. The injury of endothelial cells, instability of the macrocirculation, disturbance of blood coagulation, and activation of peroxide all lead to disturbance of the microcirculation (Ait-Oufella et al., 2015; Ratiani et al., 2015). In early stages of septic shock, the parasympathetic effect of LPS leads to blood flow redistribution and hemodilution and further reduces the local blood flow in the spleen and kidney. However, resveratrol treatment was shown to partially recover whole blood viscosity and local blood flow while also increasing the white blood cell content in the peripheral blood (Wang et al., 2018a). A meta-analysis of an animal model of sepsis with resveratrol intervention suggested that resveratrol can reduce the sepsis-induced inflammatory response by reducing TNF-α, MDA, and IL-6 levels; increasing IL-10 levels; and improving mean arterial pressure, thus improving the microcirculation (Zhou et al., 2019).

Hypovascular reactivity often occurs in the late shock stages during sepsis development. This is an important factor contributing to microcirculation disturbance and tissue hypoperfusion and further leads to multiple organ injury and dysfunction. Xu-Qing Wang et al. (2017) showed that resveratrol could maintain better mean arterial pressure in rats with LPS-induced sepsis. *In vitro* experiments revealed that resveratrol enhanced the vascular response of mice to LPS challenge through the RhoA-ROCK-MLCP signal pathway. Zhang et al. (2019) further showed that resveratrol dose-dependently inhibited the upregulation of eNOS expression by Rac-1 and HIF-1α, improved hemodynamics, reduced the decrease of hepatic and renal blood flow, and enhanced the vasodilation response of septic shock rats. Similarly, resveratrol decreased leukocyte/platelet adhesion and E-selectin/ICAM-1 expression, and increased SIRT-1 expression in obese septic mice; the same changes were found in human umbilical vein endothelial cells treated with resveratrol *in vitro* suggesting that resveratrol can reduce microvascular inflammation by increasing the expression of SIRT-1 (Xianfeng Wang et al., 2015).

### Immune System

In addition to the excessive inflammatory response represented by the overproduction of inflammatory mediators, the body enters a complex state of immune dysfunction during sepsis development and progression. This is characterized by reduced anti-infective immune defense abilities that are reflected in the decreased phagocytic and bactericidal activities and inhibition of antigen-presentation function (Kumar 2020). As innate immunomodulatory cells, macrophages play an important role during sepsis. Several studies have shown that resveratrol can regulate the activity of macrophages, suggesting a protective effect against immune dysfunction in sepsis.

Ma et al. (2017) demonstrated that resveratrol can inhibit macrophage activation by inhibiting the expression of mir-155 and upregulating cytokine signal transduction inhibitor 1. Resveratrol could also reduce the expression levels of the pro-inflammatory factors TNF-α and IL-6. This may be due to its inhibition of the phosphorylation of mitogen-activated protein kinases (MAPKs) and STAT1/3. Moreover, resveratrol can inhibit TRAF6 expression and the ubiquitination of macrophages induced by LPS while also weakening the TLR4-TRAF6, MAPK, and Akt pathways induced by LPS (Jakus et al., 2013). Moreover, resveratrol was reported to inhibit the expression of cyclooxygenase-2 induced by NF-κB and LPS, which was also related to the inhibition of TRIF-TBK1-RIP1 signaling (Youn et al., 2005). Sebai et al. (2010) confirmed that resveratrol may play an antioxidant role through a TRIF-dependent pathway.

In addition, Wang et al. (2020) showed that resveratrol limited the activation of important signal molecules (PLD, SphK1, ERK1/2, and NF-κB) stimulated by LPS at different time points during sepsis induction and progression. In the early stage of sepsis (within 1 h), resveratrol reduced cytokine production by inhibiting PLD and downstream NF-κB and ERK signaling. Throughout the course of the disease (exposure for 4 h or more), resveratrol was able to reduce MyD88-related autophagy, although the direct relationship between the two remains unclear. Resveratrol has also been shown to improve lymphocyte DNA damage in septic rats through antioxidation effects (Aydın et al., 2013).

### Gastrointestinal Tract

*In vivo* animal studies have shown that polyphenol extracts can reduce the severity of colitis by modifying various intracellular signal cascades in the intestinal epithelium and exhibiting anti-inflammatory effects (Romier et al., 2009). Similarly, resveratrol improved ileal smooth muscle reactivity in septic rats (Gacar et al., 2012).

### Adrenal Gland

Endotoxins lead to adrenal oxidative stress and excessive production of NO, which causes adrenocortical dysfunction (Chang-Nan Wang et al., 2015). Resveratrol treatment significantly inhibited iNOS expression, NO production, and peroxynitrite formation induced by endotoxemia and reduced LPS-induced adrenal oxidative stress, as evidenced by a decrease in MDA and an increase in various antioxidant biomarkers (T-AOC, CAT, and SOD activity). In addition, resveratrol (Duan et al., 2016), as an agonist of SIRT-1, reversed the LPS-induced downregulation of the adrenocorticotropin receptor and SIRT-1 as well as the weak adrenocortical response to corticotropin. Resveratrol also protects against the adrenocortical insufficiency associated with endotoxemia by inhibiting oxidation/nitrification stress. These findings support the therapeutic potential of resveratrol in alleviating adrenocortical dysfunction caused by oxidative/nitrification injuries in sepsis.

## Clinical Trials

Resveratrol is currently being explored in hundreds of clinical trials involving the nervous, respiratory, and endocrine systems as well as other domains.

Resveratrol supplementation may improve glycated hemoglobin in the short term in the clinical management of diabetes mellitus (Zeraattalab-Motlagh et al., 2021). Resveratrol also improves blood sugar control and lowers blood pressure (Nyambuya et al., 2020). In addition, resveratrol supplementation has been found to significantly reduce C-reactive protein levels in patients with type 2 diabetes (Hosseini et al., 2020).

Meta-analyses of patients with metabolic syndrome and related disorders have yielded seemingly contradictory results (Asgary et al., 2019; Akbari et al., 2020; Tabrizi et al., 2020). Therefore, more randomized controlled trials (RCTs) are needed to supplement and validate these findings in the future. In a meta-analysis of obese patients, resveratrol intake significantly reduced body weight, BMI and fat mass, and significantly increased lean body mass but did not affect leptin and adiponectin levels (Tabrizi et al., 2020). In addition, a meta-analysis involving 11 clinical studies showed that resveratrol supplementation was effective in reducing alveolar bone loss and preventing the clinical development of periodontal disease (Andrade et al., 2019). Regular resveratrol supplementation also demonstrated a positive effect on bone mineral density in postmenopausal women (Wong et al., 2020). In patients with polycystic ovary, 1,500 mg of resveratrol per day significantly reduced ovarian and adrenal androgen levels (Banaszewska et al., 2016). Resveratrol supplementation also showed effectiveness against a number of cancers including breast cancer (Zhu et al., 2012), liver cancer (Howells et al., 2011), and colorectal cancer (Patel et al., 2010). These results need to be validated in the future through larger RCTs. There are currently no trials available discussing the potential of resveratrol in sepsis treatment; therefore, further exploration is needed in the future.

## Safety

Resveratrol is divided into cis and trans structures, with the more stable trans structure found in nature. Due to differences in bioavailability and pharmacokinetics, current studies suggest that oral resveratrol reduces the level of lipid peroxidation in the small intestine and colon due to LPS-induced sepsis but has no effect on inflammatory markers (Larrosa et al., 2011), which suggests that the optimal route of administration should be selected according to the bioavailability and target organ.

Hebbar et al. (2005) administered resveratrol to CD rats at high doses. This resulted in varying degrees of dehydration, dyspnea, nephrotoxicity, and increased liver enzymes in serum indicating that resveratrol has a certain degree of toxicity at high doses. Johnson et al. (2011) confirmed that high-dose resveratrol can increase liver bilirubin levels, demonstrating subchronic oral toxicity. However, the doses tested in these studies are much higher than the current clinical dose (500–1,000 mg/day). In addition, the oral absorbance of resveratrol was estimated to be at least 75%; however, due to the rapid and extensive metabolism, biological availability is poor (Ait-Oufella et al., 2015). Therefore, the bioavailability of resveratrol remains a major concern for the development of medical products and technology. Scientists have been exploring updated *trans*-resveratrol delivery mechanisms to improve solubility and bioavailability, including methylated resveratrol analogs (Kang et al., 2014), resveratrol particle system (Peng et al., 2010), and vesicle system (Pangeni et al., 2014) carriers.

## Prospects for the Future

Resveratrol exerts a variety of sepsis-related protective mechanisms. The therapeutic potential of resveratrol has attracted the attention of researchers as sepsis remains a global problem. Many studies have been carried out in sepsis cell cultures and animal models; however, numerous hurdles still need to be overcome so that resveratrol could be utilized clinically. These hurdles include bioavailability, dose optimization, and side effect reduction. Currently, large-scale, clinical application of resveratrol requires more pre-clinical and clinical studies. Also, the preparation process of resveratrol somewhat restricts its development and application. For example, the bioactivity of chemically synthesized resveratrol is not as good as that of natural products, but the cost and purity of natural products pose a challenge. Some of these hurdles could be tackled via modern drug development technologies. For example, precursor drugs could be synthesized using nanomaterial coating while also chemically modifying the active ingredient, resveratrol. This would allow its release across the intestinal tract to specific target organs improving its bioavailability and providing the possibility to improve the research status of resveratrol and further their clinical application. Thus, the comprehensive mechanism and clinical application of resveratrol remain important topics of exploration in future research.
